# Time Perception in Cocaine-Dependent Patients

**DOI:** 10.3390/brainsci12060745

**Published:** 2022-06-06

**Authors:** Giovanna Mioni, Naomi Sanguin, Graziella Madeo, Stefano Cardullo

**Affiliations:** 1 Dipartimento di Psicologia Generale, Università di Padova, 35131 Padova, Italy; naomi.sanguin@studenti.unipd.it; 2 Novella Fronda Foundation, 35121 Padova, Italy; graziemadeo@gmail.com (G.M.); stefano.cardullo@gallimbertipartners.com (S.C.)

**Keywords:** time perception, stimulant dependent, time bisection task

## Abstract

The involvement of the dopamine system in modulating time perception has been widely reported. Clinical conditions (e.g., Parkinson’s disease, addictions) that alter dopaminergic signaling have been shown to affect motor timing and perceived duration. The present study aimed at investigating whether the effects of chronic stimulant use on temporal processing are time-interval dependent. All participants performed two different time bisection tasks (480/1920 ms and 1200/2640 ms) in which we analysed the proportion of long responses for each stimulus duration as well as an index of perceived duration and one of sensitivity. Regarding the proportion of long responses, we found no differences between groups in either time bisection task but patients had more variable results than controls did in both tasks. This study provides new insight into temporal processing in stimulant-dependent patients. Regardless of the time interval tested, the results showed comparable temporal ability in patients and controls, but higher temporal variability in patients. This finding is consistent with impairment of frontally-mediated cognitive functions involved in time perception rather than impairment in time processing per se.

## 1. Introduction

Time is a fundamental dimension for adequately dealing with most of our everyday activities. Traditionally, time processing has been explained accordingly to the Internal Clock Model [1,2] hypothesising a pacemaker–accumulator system. The model is composed by three stages, namely clock, memory and decision stages, and predicts that subjective perception of time depends on the number of pulses produced regularly by a pacemaker (clock stage) and stored into the accumulator. Neuroimaging studies have identified the cortico-striatal network as the neural basis of the internal clock [3]; in particular, accurate temporal processing requires an optimal level of dopaminergic function in cortico-striatal circuits in order to control clock speed [4]. The first investigations of the biological substrates of the internal clock model used pharmacological manipulations and provided considerable support for involvement of the dopaminergic system in temporal processing [4]. Indeed, the dopaminergic system has been identified as a critical neural system for the processing of time and for the sensory-motor integration that generates the intricate and precisely controlled motor actions [5,6]. Dopamine antagonists (e.g., haloperidol) that affect the mesostriatal dopamine system slow down the clock rate in healthy subjects [7], whereas animals and humans under the acute influence of psychostimulants overestimate time as a result of speeding up the clock rate [5]. Support for the involvement of the dopaminergic system in time processing also comes from studies with Parkinson’s disease (PD) patients, which is a neurodegenerative disorder characterised by loss of dopamine-producing neurons [8]. PD patients tested off medication report deficits in motor timing and in perceived duration but show adequate temporal abilities when medicated with L-dopa [9,10]. Nevertheless, the magnitude of temporal dysfunction depends on the heterogeneity of PD (both clinical and cognitive characteristics) and the type of temporal task used [9].

Dysregulation of the dopaminergic system is also related to chronic use of drugs such as stimulants [11]. Several cognitive dysfunctions have been reported in stimulant-dependent individuals [12], however, it is not known whether these individuals have fundamental problems with temporal processing. An answer to this question is of interest because various higher-order cognitive functions are dependent on intact temporal processing [5]. Previous research in rats showed cocaine-induced temporal overestimation [13,14] but the few studies conducted with stimulant-dependent users reported conflicting results. Mohs et al. [15] revealed relatively shortened temporal production following acute methamphetamine injection after 1, 2 and 3 h, reflecting a continuous acceleration of cognitive processes. Wittmann et al. [16] showed that stimulant-dependent patients presented deficits in perceptual and motor timing in temporal intervals around 1–2 s. Zhang et al. [17] reported group differences in a time reproduction task for short-term abstinence but no differences in a time discrimination task, concluding that alteration of time perception in methamphetamine-dependent patients is task-specific and dose-dependent.

We were interested in whether we would find specific effects of chronic stimulant use that depended on time intervals. Previous studies have emphasised that temporal processing of short intervals (less than 1 s) is more automatic and is frequently linked with motor control, whereas temporal processing of longer intervals (over 1 s) requires the support of additional cognitive resources [18,19]. The time bisection task has been used extensively in the study of temporal processing; it is also considered one of the purest measures of time perception. We predicted temporal overestimation in stimulant-dependent patients consistent with the effect of a stimulant on clock speed [5]. We also predicted higher temporal variability in stimulant-dependent patients compared to control due to more variable temporal representation in patients caused by compromised cognitive abilities involved in temporal processing [19,20,21]. To test our hypothesis, we employed a time bisection task, in which, participants are instructed to categorize temporal intervals as being more similar to the short or to the long standard intervals previously learned [22]. The time bisection task has been extensively used in the study of temporal processing and it is also considered one of the most appropriate measures of time perception, in particular, when clinical populations are involved because it reduced the motor component [23,24]. (Notably, participants were not methamphetamine-dependent patients but healthy participants tested after they had received 10 mg of methamphetamine, 100 mg of secobarbital or placebo on separate days).

## 2. Materials and Methods

### 2.1. Participants and Procedure

Twelve patients and 20 stimulant-dependent users were included in the study (Table 1).

Twelve patients (1 female; mean age (SD) = 48.17 (10.05) years; range 28–62 years old) and 20 controls (14 male; mean age (SD) = 45.72 (10.12) years; range 26–60 years old) were included in the study. Patients were eligible for the study if they met the criteria for stimulant use disorder, as determined by interview, as per the Diagnostic and Statistical Manual of Mental Disorders, 5th edition, and by consensus of a team of experienced clinicians. Nine patients reported having only used cocaine and three reported to be mainly cocaine-dependent but occasional heroin users. Stimulant-dependent participants reported to have used a stimulant for the first time at the mean age of 19.21 years (SD = 5.13; range 14–31 years) and to have used stimulants for an average of 28.17 years (SD = 10.07; range 7–43 years). Eight patients reported that they used a stimulant daily and four patients reported that they used a stimulant one or more times a week, in particular during weekends. Stimulant-dependent participants reported to have stopped consuming stimulants upon entering the community and that they were not under the effect of a stimulant at the time of testing (mean 119 days between last consumption and day of testing).

Patients were all residents of “Noi Associazione Famiglie Padovane Contro l’Emarginazione Onlus”, Padova (Italy), and were tested in a quiet room at the residential centre; controls were tested in their own home in the area of Padova. The experiments gave particular attention to controlling the environmental conditions (residential and home) to equate the different testing environments. All clocks were removed, and we asked participants to turn off their cell-phones. During the tasks, participants were seated at a distance of approximately 60 cm in front of a 15-inch PC monitor screen. PsychoPy [25] (Peirce, Gray, Halchenko, Britton, Rokem & Strangman, 2011) was used to program and run the experiments. All participants were tested during one experimental session that lasted approximately 60 min. All participants gave written consent to participate in the study. The study was conducted in accordance with the Helsinki Declaration (59th WMA General Assembly, Seoul, 2008); the ethics committee of the Department of General Psychology (protocol number 3817) approved the procedure and we also received approval by the director of the residential centre to test patients.

### 2.2. Time Bisection Task

All participants performed two different time bisection tasks: 480/1920 ms and 1200/2640 ms, with the order being randomised between participants. Each timing task started with the learning phase in which participants were required to memorize two standard durations: 480 ms (short anchor) and 1920 ms (long anchor) for the 480/1920 ms task and 1200 ms (short anchor) and 2640 ms (long anchor) for the 1200/2640 ms task. The standard durations were presented 10 times in each task. After the learning phase, participants were instructed to estimate whether the probe durations were closer to the “short anchor” or the “long anchor” previously memorized. Participants performed three blocks and within each block, each probe duration was presented 10 times in random order. Temporal intervals were marked by a grey circle presented (two cm diameter) at the centre of the computer screen. In the 480/1920 ms condition, the short anchor duration was 480 ms and the long anchor was 1920 ms; the probe durations were 480, 720, 960, 1200, 1440, 1680 and 1920 ms. In the 1200/2640 ms condition, the short anchor duration was 1200 ms and the long anchor was 2640 ms; the probe durations were 1200, 1440, 1680, 1920, 2160, 2400 and 2640 ms. Participants were asked to respond with their left and right index fingers, and response keys were counterbalanced between participants.

### 2.3. Statistical Analyses

For each time bisection task, a seven-point psychometric function was traced for each participant, plotting the seven comparison intervals on the x-axis and the probability of responding “long” on the y-axis. The cumulative normal function was fitted to the resulting curves. We analysed the proportion of long responses for each stimulus duration separately for the two anchor conditions 480/1920 ms and 1200/2640 ms. The data were included in two separate repeated measure analyses of variance with Group (stimulant dependent and controls) as the between-subjects factor and Temporal interval (480/1920 ms and 1200/2640 ms for the two conditions) as the within-subject factors. All significant effects were followed by post hoc analyses performed with a Bonferroni correction to reduce the Type I error rate, and the effect size was estimated with partial eta squared (η^2^p).

We also calculated two indices, one that defines perceived duration and one for temporal sensitivity. The first was the bisection point (BP), that is, the stimulus duration at which participants responded “short” or “long” with equal frequency. An observed shift of the BP can be interpreted as an indicator of differences in perceived duration, with smaller BP values meaning longer perceived durations. The second dependent variable was the Weber ratio (WR), which is based on one standard deviation (SD) on the psychometric function, specifically, the WR is the SD divided by the BP. The WR is a measure of temporal sensitivity; smaller values indicate more sensitive timing. Separate t-tests were conducted on BP and WR, and we estimated effect size with Cohen’s d. Considering the small sample size and variety in the number of subjects of the two samples, we also adopted a bootstrap approach for the calculation of the 95% confidence interval of each estimate. For each analysis of variance and t-test we proceeded randomly resampling the group assignment without replacement (B = 1000). We computed the 95% confidence intervals on the resulted distributions of 1000 F’s and t’s obtained after each resampling. An observed estimate outside the bootstrapped CI intervals would represent a robust result not due to the random resampling process. Analyses were performed using R [26] and Jamovi [27].

## 3. Results

### 3.1. 480/1920. ms Condition

**Figure 1:**
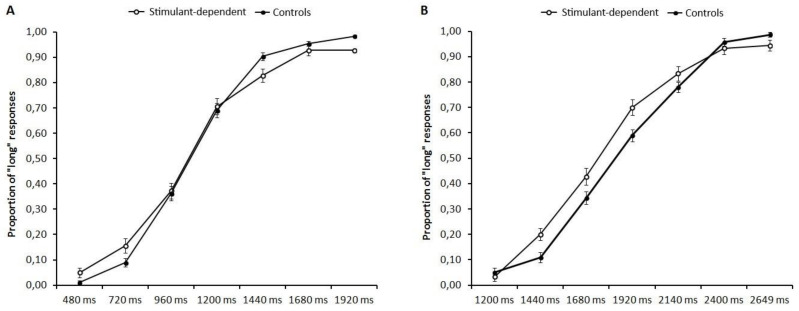
Proportion of “long” responses as a function of temporal intervals for (**A**) Short (480/1920 ms) and (**B**) Long (1200/2640 ms) anchors. Error bars indicate 95% confidence interval.

Regarding the proportion of long responses, we found significant main effects for Temporal interval (F(6,180) = 267.08; bootstrapped 95% CI: 199.13–224.5, *p* < 0.001, η^2^p = 0.90), indicating that participants increased the proportion of long responses as the duration of the probe interval increased. No main effect of Group (F(1,30) = 0.01; bootstrapped 95% CI: 0.00–13.46, *p* = 0.918, η^2^p = 0.01) or interaction between Group and Temporal interval (F(6,180) = 1.12; bootstrapped 95% CI: 0.10–2.35, *p* = 0.354, η^2^p = 0.04) was found (Figure 1A).

**Figure 2:**
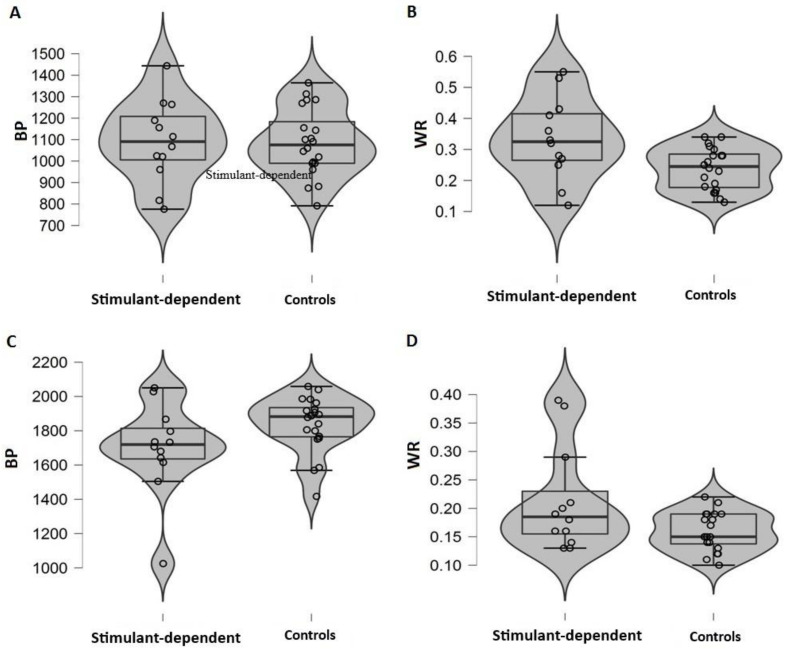
(**A**,**C**) Bisection Point (PSE) and (**B**,**D**) Weber ratio (WR) for stimulant dependent and controls. Each dot represents a participant. The thicker line represents the median and the box represents the interquartile range. The grey area represents the kernel density estimation to show the distribution shape of the data. Wider sections of the violin plot represent a higher probability that members of the population will take on the given value; the skinnier sections represent a lower probability.

When data were analysed in terms of BP, we observed a similar perceived time in patients and controls (t(30) = 0.094; bootstrapped 95% CI: −1.91–2.13, *p* = 0.925, d = 0.03; stimulant dependent = 1091(191), controls = 1085(157)). However, patients showed more variability (WR) than controls did (t(30 =2.721; bootstrapped 95% CI: −1.91–2.18, *p* = 0.011, d = 0.99; stimulant dependent = 0.33(0.13), controls = 0.24(0.07)) (Figure 2A,B).

### 3.2. 1200/2640. ms Condition

Regarding the proportion of long responses, we found a significant main effect for Temporal interval (F(6, 180) = 290.32; bootstrapped 95% CI: 203.82–227.27, *p* < 0.001, η^2^p = 0.91), indicating that participants increased the proportion of “long” responses as the duration of the probe interval increased. No main effect of Group (F(1, 30) = 1.13; bootstrapped 95% CI: 0.00–12.93, *p* = 0.295, η^2^p = 0.04) or interaction between Group and Temporal interval (F(6, 180) = 2.05; bootstrapped 95% CI: 0.11–2.11, *p* = 0.062, η^2^p = 0.06) was found (Figure 1B).

When data were analysed in terms of BP, we observed similar perceived time in patients and controls (t(30) = 1.82; bootstrapped 95% CI: −2.04–1.88, *p* = 0.078, d = 0.67; stimulant dependent = 1698(265), controls = 1836(163)). However, patients showed more variability (WR) than did controls (t(30) = 2.418; bootstrapped 95% CI: −1.91–2.14, *p* = 0.022, d = 0.88; stimulant-dependent = 0.21(0.09), controls = 0.16(0.03)) (Figure 2C,D).

## 4. Discussion

Stimulant dependent users show altered function in prefrontal and striatal dopaminergic circuits [12]; since these neural circuits are also involved in temporal processing and motor timing [3], we hypothesised temporal misperception in stimulant-dependent participants compared with controls. Although we did not observe group differences in stimulant-dependent patients compared with controls in the 480/1920 ms condition, we did observe a tendency toward temporal overestimation when patients performed the 1200/2640 ms condition. Temporal processing of short intervals is frequently associated with motor control because automatic movements are typical of sub-second durations, whereas the processing of longer intervals requires the support of additional cognitive resources [18]. For studies that use short and long intervals, investigators found that the cerebellum and basal ganglia seem to be involved in either short or long intervals, whereas prefrontal regions seem to be more involved in processing long temporal intervals [3,18]. These patterns of results suggest that prefrontal areas may mediate time perception by means of additional cognitive functions such as attention and working memory [19].

The lack of differences between groups in both time perception temporal ranges may be related to the duration of abstinence in stimulant-dependent patients. Compared with that reported in previous studies, patients in our sample reported being abstinent for a longer time (our sample 127 ± 176 days; [16] methamphetamine or cocaine users 27.7 ± 5.6 days; [17] methamphetamine users 69.74 days ± 35.95). Despite the long-term effects on dopamine availability in cocaine abusers [22,28], it is possible that the neurobiological changes that occur during prolonged abstinence (>3 months) may be enough to overcome the impairment in time processing. Notably, temporal overestimation in rats was observed when they were tested under the effects of a stimulant (cocaine) and tested with much longer temporal intervals [13]. Using a time production task (12 s) and a time bisection task (2–8 s), Cheng et al. [13] showed that cocaine shifted the psychophysical functions leftward relative to control conditions indicating temporal overestimation in both temporal tasks. In contrast, ketamine produced no change in time perception on either procedure. Later, Cheng and colleagues [14] showed that the effect of cocaine on timing also depends on dosage and training (number of training sessions before drug injection). Only rats that have received a minimum amount of training (less than 30 sessions) prior to drug administration displayed a leftward shift in their timing functions indicating temporal overestimation. Similarly, patients with PD showed temporal modification when tested on or off medication [29], indicating a relationship between temporal modification and time and dose administration. This finding is also confirmed by previous studies conducted with stimulant-dependent patients [15,17]. Importantly, Zhang et al. [17] showed that motor timing (tested with a time reproduction task, temporal range between 1 and 5 s), but not perceptual timing (tested with two time discrimination tasks, 200–800 ms and 1400–1600 ms), was altered in meth dependents, which persisted for at least 3 months of abstinence. The authors concluded that time perception alteration in meth dependents is task-specific and dose-dependent [17].

In our study, we observed higher variability in stimulant-dependent participants than in controls. Similarly, temporal dysfunctions in patients with brain lesions [19] as well as in healthy [20] and pathological aging [21] patients have been mainly related to deficits in cognitive functions involved in temporal processing (i.e., working memory, attention, and executive functions) rather than to an impairment in time perception caused by a compromised internal clock. The higher variability often observed in patients has been interpreted as difficulty in maintaining a stable representation of duration caused by frontally-mediated dysfunction [19]. Considering the executive dysfunctions observed in stimulant-dependent patients [12,30], we speculate that in patients with prolonged abstinence, group differences are mainly caused by impaired frontally-mediated cognitive functions involved in time perception rather than impairment in time processing per se. However, future studies are needed to test this hypothesis.

A limitation of the present study is the sample size. We acknowledge that the two groups are quite small, but we believe that our study can still provide interesting insights into the understanding of temporal dysfunction in stimulant-dependent patients. Few studies have been conducted to investigate the effect of a stimulant on time perception [16,17] and this is the first to include patients who use cocaine. Investigating temporal processes is of great importance considering that adequate temporal abilities are linked to many cognitive functions.

The present study adds new insight into temporal processing in stimulant-dependent patients. The results showed comparable temporal ability in patients and controls, suggesting a similar pattern of temporal processing in both groups, but higher temporal variability in patients, consistent with impaired frontally-mediated cognitive functions involved in time perception. Future studies should consider taking into account not only the duration of abstinence but also the severity of patients’ cognitive dysfunction.

## Figures and Tables

**Table 1:** Description (mean and standard deviation) of stimulant-dependent and control participants.

	Stimulant-Dependent n° 12	Controls n° 20	
	M (SD)	M (SD)	*t*
Age	47.8 (10.1)	46.5 (10.2)	0.35
Education	9.25 (2.26)	11.3 (2.90)	2.09 *
Gender (M)	11	14	
Type of drug used			
Only cocaine	9	-	
Occasionally also heroin	3	-	
Duration of current abstinence (days)	127 (176)	-	
Age of the first assumption	19.21 (5.13)	-	
Frequency of assumption	8 daily	-	
	4 weekends	-	

Note: * *p* < 0.05.

## Data Availability

The data that support the findings of this study are available from the corresponding author upon request.
